# What is gratitude research missing? Bridging the gap between conceptualization, measurement, and intervention

**DOI:** 10.3389/fpsyg.2026.1910738

**Published:** 2026-10-15

**Authors:** David del Rosario-Gilabert, Mamen Regalado-Reyes, Neus Vila-Brunet

**Affiliations:** Instituto de Neurociencia Avanzada de Barcelona (INAB), Barcelona, Spain

**Keywords:** gratitude, gratitude interventions, gratitude measurement, gratitude neuroscience, positive psychology

## Abstract

Does contemporary gratitude research investigate the full phenomenon described by current gratitude theories? Gratitude has become a prominent construct in positive psychology, with research linking it to well-being, resilience, prosociality, and mental health. As theoretical perspectives have expanded, however, empirical approaches appear not to have broadened to the same extent. This purposive Mini review examines gratitude research across conceptualization, measurement, intervention, physiology, and neuroscience. Contemporary theories and measures increasingly represent gratitude as a multidimensional phenomenon encompassing interpersonal, existential, contemplative, embodied, and ecological dimensions. By contrast, dominant and influential intervention paradigms continue to emphasize reflection on benefits and benefactors, while much of the neuroscience literature reviewed here operationalizes gratitude through interpersonal, appraisal-based, and reciprocity-related scenarios. These differences suggest a conceptual–operational mismatch: relatively broad understandings of gratitude are often investigated through more narrowly focused empirical approaches. Consequently, existing findings may illuminate interpersonal and benefit-focused gratitude more fully than broader forms of grateful experience. We conclude by proposing directions for developing ecologically valid, embodied, and relational methodologies capable of investigating a wider range of gratitude experiences.

## 1 Introduction

What exactly is gratitude? Is it simply the appreciation felt after receiving kindness from others, or can it also emerge through awe, nature, contemplation, or suffering itself? Over the past two decades, gratitude has become a prominent construct in psychology (McCullough et al., 2001; McCullough et al., 2002), contemplative and transpersonal research (Hlava et al., 2014; Büssing et al., 2018), and neuroscience (Fox et al., 2015b; Yu et al., 2017, 2018). Psychological research has linked gratitude to well-being and mental health (Wood et al., 2010; Jans-Beken and Wong, 2021), prosociality and relational functioning (McCullough et al., 2001; Algoe, 2012), and resilience in the face of adversity (Jans-Beken and Wong, 2021). Despite this growing multidisciplinary interest, gratitude remains conceptually heterogeneous (Wood et al., 2010; Jans-Beken and Wong, 2021).

Early psychological models primarily defined gratitude as an interpersonal emotion arising when individuals recognize that they have received a valuable and intentionally provided benefit from another person (McCullough et al., 2001, 2002). Within this framework, gratitude became closely associated with reciprocity, prosociality, and relationship maintenance (McCullough et al., 2001; Algoe, 2012). Later perspectives expanded this understanding by proposing that gratitude may also reflect a broader orientation toward life and emerge through awe, nature, spirituality, embodiment, interconnectedness, and adversity (Wood et al., 2010; Hlava et al., 2014; Büssing et al., 2018; Jans-Beken and Wong, 2021). Gratitude consequently came to encompass not only responses to received benefits but also forms of receptivity, presence, and engagement with existence itself.

Several terms require clarification. In this review, embodied gratitude refers to grateful experience in which bodily sensations, autonomic regulation, and felt engagement with the immediate situation constitute part of how gratitude is experienced, rather than merely accompanying it as physiological correlates. Ecological gratitude refers to gratitude directed toward, or arising through, nature, the more-than-human world, and ecological interconnectedness. It is distinct from ecologically valid methods, which capture experience in everyday settings rather than exclusively through artificial or decontextualized experimental tasks. Finally, relational participation is introduced here as a phenomenological reframing rather than an established theoretical construct. It describes gratitude as experienced through participation in relationships—with other people, communities, nature, or life more broadly—rather than solely through appraisal of a discrete benefit received from an identifiable benefactor.

This conceptual broadening is also reflected in measures encompassing dispositional, existential, awe-related, and transpersonal dimensions of gratitude (Watkins et al., 2003; Hlava et al., 2014; Büssing et al., 2018; Jans-Beken and Wong, 2021). However, within the literature reviewed here, influential interventions frequently use reflective exercises focused on positive events, benefits, or benefactors, while neuroscientific studies commonly employ helping scenarios and reciprocity-based exchanges (Emmons and McCullough, 2003; Seligman et al., 2005; Fox et al., 2015b; Yu et al., 2017, 2018). This suggests a possible conceptual–operational mismatch: gratitude is theorized and measured in relatively broad terms but often induced and investigated through more narrowly focused empirical paradigms.

This purposive Mini Review examines that mismatch across conceptual, psychometric, interventional, physiological, and neuroscientific research. It considers how influential operationalizations shape what gratitude research can explain and identifies directions for investigating a broader range of grateful experiences.

## 2 Review approach

This article adopts a purposive Mini Review approach to examine how gratitude has been conceptualized, measured, induced, and investigated across psychological, physiological, and neuroscientific research. It is conceptually integrative rather than systematic and does not seek to identify every publication, estimate the prevalence of particular approaches, or compare their relative frequency.

Literature searches were conducted between 28 January and 5 June 2026 using combinations of the following terms: “gratitude,” “gratitude intervention,” “gratitude scales,” “gratitude protocol,” “reciprocity and gratitude,” “neuroscience and gratitude,” “holistic gratitude,” “spirituality and gratitude,” “biomarkers and gratitude,” “models and gratitude,” and “positive psychology and gratitude.” Searches were conducted using Google Scholar, Web of Science, and Scopus. Publications were included when they directly informed at least one of the review’s five analytical domains—conceptualization, measurement, intervention, physiology, or neuroscience—or when they were necessary to trace the historical development of an influential paradigm. Publications were excluded when gratitude was mentioned only as a secondary variable without contributing to its conceptualization or operationalization. Foundational publications identified through prior knowledge and additional publications located through citation searching were also considered when needed to clarify the field’s historical development, relevant conceptual perspectives, or assumptions underlying influential methods.

The initial evidence matrix comprised 26 publications. Eleven additional foundational or directly relevant publications were subsequently incorporated through citation searching and targeted searches addressing conceptual or empirical areas identified during the review. This produced a final corpus of 37 distinct publications across the narrative review and supplementary intervention analysis. Formal database-level record counts were not retained because the searches were conducted for a purposive Mini Review rather than under a preregistered systematic-review protocol. The figures reported therefore concern the publications selected and examined, not the total records returned by the searches.

The publications were compared across conceptual, psychometric, interventional, physiological, and neuroscientific domains. The analysis examined how gratitude was defined, elicited, and measured; whether an identifiable benefit or benefactor was required; and which interpersonal, cognitive, experiential, bodily, relational, or neural processes were emphasized. It also considered the correspondence between theoretical conceptualizations and empirical operationalizations. Because the corpus was purposively selected, observations about influential paradigms apply only to the literature examined and should not be interpreted as quantitative estimates of their prevalence across the wider field.

## 3 Conceptualizations of gratitude

### 3.1 Benefit-appraisal and reciprocity traditions

Early experimental research conceptualized gratitude as a response to an interpersonal benefit. Tesser et al. (1968) proposed that gratitude depends on recipients’ appraisals of the benefactor’s intention to help, the cost incurred by the benefactor, and the value of the benefit to the recipient. All three factors predicted imagined gratitude in hypothetical scenarios. Lane and Anderson (1976) subsequently found that intention and outcome value were integrated through an averaging process when participants judged how grateful a recipient should feel. Together, these studies established intention, benefactor cost or effort, and benefit value as central experimental dimensions of gratitude-eliciting situations.

This work made benefit appraisal experimentally tractable but examined gratitude in specific ways. Both studies used hypothetical benefactor–beneficiary scenarios; Lane and Anderson (1976) investigated normative judgments about another person’s gratitude rather than directly experienced emotion, while Tesser et al. (1968) combined gratitude and indebtedness ratings because they were strongly correlated. Subsequent research has distinguished gratitude—a positively valenced response to another’s goodwill—from indebtedness and obligation, which are more closely associated with pressure to repay. These foundational studies therefore primarily illuminate the cognitive appraisal of interpersonal benefits.

Benefit-appraisal research developed alongside accounts of reciprocity. The norm of reciprocity proposes that receiving a benefit creates an expectation that assistance should be returned to the benefactor (Gouldner, 1960). Evolutionary and psychological accounts distinguish such direct reciprocity from upstream reciprocity, in which receiving help increases assistance to third parties (Nowak and Roch, 2007). Gratitude can motivate costly helping beyond the original benefactor (Bartlett and DeSteno, 2006), while relational accounts propose that it also helps individuals identify responsive partners and build enduring relationships (Algoe et al., 2008; Algoe, 2012).

Naturalistic findings nevertheless show that verbal expressions of thanks, reciprocal behavior, and felt gratitude are not interchangeable. Across two field studies, Fox et al. (2015a) found that high-effort door-holding increased the likelihood of verbal thanks. In Study I, however, door-holding effort did not increase participants’ willingness to complete a survey for another person, an indicator of upstream helping. In Study II, high-effort door-holding increased direct helping toward the door-holder, measured by whether participants returned to help pick up dropped pens. Importantly, verbal thanks did not predict either form of subsequent helping, and participants’ subjective experience of gratitude was not directly measured. These behaviors may reflect gratitude, but they may also be influenced by politeness, perceived helpfulness, indebtedness, empathy, or reciprocity norms. The study therefore demonstrates the value of naturalistic behavioral paradigms while also illustrating why verbal thanks and reciprocal helping should not be treated as direct measures of felt gratitude.

Fox, Shah and Brownell’s gift-space model extends this tradition by examining how benefactor effort and recipient need jointly shape gratitude, including between strangers and during hardship (Fox et al., 2026). Their findings indicate that both effort and need are strongly associated with gratitude and that gratitude may coexist with positive and negative emotions. Although the model broadens the situations considered, it retains a benefactor–benefit structure. This provides the point of contrast for the existential, experiential, and transpersonal perspectives discussed next.

### 3.2 Existential, experiential, and transpersonal gratitude

Later perspectives extended gratitude beyond responses to discrete interpersonal benefits. Emmons and Shelton (2002) described gratitude as a worldview through which life itself may be perceived as gift-like, while Wood et al. (2010) conceptualized it as a broader life orientation involving the tendency to notice and appreciate positive aspects of existence. Similarly, Watkins et al. (2003) identified the appreciation of simple pleasures as one component of dispositional gratitude, alongside a sense of abundance and appreciation of others. This form of appreciation does not necessarily depend on an intentional benefactor or a clearly defined benefit.

Gratitude has also been examined in relation to spirituality and religious worldviews. Spiritual or religious frameworks may provide interpretive contexts in which positive experiences, relationships, and life more broadly are understood as gifts (Emmons and Shelton, 2002; Emmons and Kneezel, 2005). From this perspective, gratitude may be directed toward God, a transcendent source, life as a whole, or circumstances that are not attributable to an identifiable human benefactor. Research has also associated dispositional gratitude and gratitude practices with subjective well-being, although gratitude overlaps conceptually and empirically with appreciation, spirituality, and positive affect (Emmons and McCullough, 2003; Watkins, 2013).

Existential gratitude further broadens the construct by referring to the capacity to experience gratitude amid adversity, suffering, and limitation, rather than only in response to positive circumstances (Jans-Beken and Wong, 2021). Within second-wave positive psychology, this perspective connects gratitude with meaning-making, resilience, and tragic optimism (Wong, 2011; Jans-Beken and Wong, 2021).

Transpersonal and awe-related accounts emphasize interconnectedness, transcendence, spirituality, beauty, wonder, and self-transcendent experience (Hlava et al., 2014; Büssing et al., 2018). Together, these perspectives suggest that gratitude may arise through pathways other than the appraisal of a discrete benefit, including appreciation of ordinary experience, spiritual interpretation, meaning-making amid adversity, contemplative awareness, and engagement with nature. Table 1 summarizes the distinction between reactive or appraisal-based gratitude and broader dispositional, existential, and transpersonal orientations.

**Table 1:** Comparative dimensions of reactive and dispositional forms of gratitude.

Gratitude dimensions	Reactive/appraisal-based gratitude	Dispositional/existential gratitude
Core definition	Gratitude arising in response to perceived benefits or positive events	Gratitude as an enduring orientation toward existence, life, or interconnectedness
Trigger	External positive event or benefactor	Mode of being / receptive awareness
Dependence on benefactor	High	Optional or absent
Temporal structure	Episodic and state-based	Enduring and dispositional
Orientation	Retrospective and event-focused	Present-centered and existential
Mechanism	Cognitive appraisal and emotional amplification	Receptive awareness and existential attunement
Relational structure	Often dyadic (benefactor–recipient)	Can be transpersonal or non-object directed
Typical interventions	Journaling, counting blessings, gratitude letters	Contemplative, meditative, philosophical, spiritual practices
Dominant research paradigm	Positive psychology interventions	Existential, contemplative, and philosophical traditions
Main outcomes emphasized	Positive affect, wellbeing, optimism	Meaning, connectedness, awareness, transcendence

## 4 Measuring gratitude

The conceptual broadening outlined in Section 3.2 is reflected in the development of measures assessing different dimensions of gratitude. The Gratitude Questionnaire–Six Item Form (GQ-6) assesses the general tendency to experience gratitude across situations (McCullough et al., 2002). The Gratitude, Resentment, and Appreciation Test (GRAT) operationalizes dispositional gratitude through appreciation of others, appreciation of simple pleasures, and a sense of abundance (Watkins et al., 2003). Thus, although both instruments assess dispositional gratitude, the GRAT explicitly includes forms of appreciation that extend beyond discrete interpersonal benefits.

Subsequent instruments operationalized additional dimensions identified in broader gratitude theories. The Gratitude/Awe Questionnaire (GrAw-7) assesses gratitude and awe in relation to beauty, nature, stillness, and moments of pause (Büssing et al., 2018). The Existential Gratitude Scale assesses gratitude in the context of suffering and adversity (Jans-Beken and Wong, 2021), while the Transpersonal Gratitude Scale captures spiritual experience, interconnectedness, and gratitude extending beyond personal or material benefits (Hlava et al., 2014).

Together, these measures translate distinct conceptualizations of gratitude into psychometric instruments, ranging from a generalized grateful disposition and appreciation to awe-related, existential, and transpersonal dimensions. Their development provides a foundation for examining a broader range of gratitude experiences. However, evidence supporting the validity of these measures does not by itself demonstrate that the corresponding states can be elicited through existing gratitude interventions.

## 5 Gratitude interventions and operationalizations

### 5.1 Influential reflective intervention paradigms

Gratitude journaling, counting blessings, gratitude letters, and benefit-recall exercises are influential approaches to gratitude cultivation (Emmons and McCullough, 2003; Seligman et al., 2005; Wood et al., 2010). These practices direct participants’ attention toward positive experiences, supportive relationships, or beneficial circumstances and encourage them to identify, record, or reflect on what they appreciate.

Gratitude journaling and counting-blessings exercises primarily cultivate gratitude through repeated reflection on positive aspects of life. Emmons and McCullough (2003), for example, asked participants to list things for which they were grateful and found benefits for several well-being outcomes, although results varied across studies and measures. Seligman et al. (2005) similarly found that a three-good-things exercise produced sustained improvements in happiness and depressive symptoms, whereas the effects of a gratitude visit were substantial but shorter-lived. Although the three-good-things exercise does not isolate gratitude from broader positive reflection, it illustrates the attentional and interpretive processes frequently used in gratitude-related interventions.

Gratitude letters and visits add an explicitly interpersonal component by asking participants to identify a benefactor and communicate appreciation for a benefit received. These practices are consistent with social-functional accounts in which gratitude helps identify responsive partners and strengthen relationships (Algoe, 2012). Their operational structure nevertheless remains centered on identifiable benefactors, benefits, and expressions of appreciation. Supplementary Table S1 compares the principal intervention paradigms discussed in this review.

### 5.2 Emerging contemplative and existential approaches

Conceptual and measurement research has broadened gratitude to encompass contemplative, awe-related, transpersonal, and existential dimensions. These perspectives emphasize receptivity, interconnectedness, spirituality, and appreciation amid adversity (Hlava et al., 2014; Büssing et al., 2018; Jans-Beken and Wong, 2021). However, these studies provide theoretical or psychometric foundations rather than evidence for interventions designed to cultivate these forms of gratitude.

Direct experimental evidence remains preliminary. Kyeong et al. (2017) examined a five-minute guided meditation in which participants focused on their mother and mentally expressed love and appreciation. Compared with a resentment condition, the gratitude induction was associated with lower heart rate and differences in brain–heart coupling and functional connectivity. Although described as gratitude meditation, the practice retained an interpersonal focus on an identifiable person.

Hirshberg et al. (2018) compared brief gratitude, breath-awareness, loving-kindness, and attention-control practices. The gratitude practice increased positive affect immediately after the intervention but was also associated with greater negative affect and perceived aversiveness following an acute stressor. These findings indicate that the effects of brief contemplative gratitude practices may depend on context and should not be assumed to be uniformly beneficial.

Existing evidence therefore provides stronger conceptual and psychometric foundations for existential, transpersonal, and awe-related gratitude than direct intervention evidence. Contemplative gratitude has received preliminary experimental examination, but interventions specifically designed to cultivate existential, embodied, or nature-based gratitude remain to be developed and systematically tested.

### 5.3 The conceptual–interventional mismatch

Taken together, the preceding evidence reveals an asymmetry between the breadth of gratitude conceptualization and the range of practices used to cultivate it. Conceptual and psychometric research encompasses gratitude as a response to interpersonal benefits, a general orientation toward appreciation, an experience of awe or interconnectedness, and a capacity sustained amid adversity (Watkins et al., 2003; Hlava et al., 2014; Büssing et al., 2018; Jans-Beken and Wong, 2021). By contrast, influential interventions reviewed here predominantly cultivate gratitude through reflection on positive events, benefits, or benefactors (Emmons and McCullough, 2003; Seligman et al., 2005). Direct experimental work on contemplative gratitude remains preliminary, while interventions specifically targeting existential, embodied, or nature-based gratitude are not represented in the reviewed corpus.

This difference matters because an intervention’s design determines which aspects of gratitude it can elicit and which outcomes can reasonably be attributed to them. Benefit-focused exercises provide evidence about reflective appreciation and interpersonal gratitude but cannot, without additional measures or comparison conditions, establish whether their findings extend to awe-related, existential, transpersonal, or ecologically oriented experiences. The issue is therefore not the validity or value of established interventions, but the scope of the phenomenon they operationalize. Table 2 summarizes the correspondence between the broader conceptualizations identified in Sections 3 and 4 and the intervention approaches examined in this section.

**Table 2:** The conceptual-interventional mismatch in gratitude research.

Dominant conceptualization of gratitude	How gratitude is theoretically framed	Typical operationalization/intervention	Implicit mechanism assumed	Main limitation/mismatch
Gratitude as emotion/affect	Short-term positive emotional response to receiving benefits	Gratitude journaling, counting blessings, gratitude lists	Cognitive appraisal and attentional reframing	Reduces gratitude to event-based positive affect
Gratitude as trait/disposition	Stable tendency to notice and appreciate positive aspects of life	Self-report scales (e.g., GQ-6), repeated reflection exercises	Personality disposition and emotional regulation	Measures enduring orientation but interventions remain episodic
Gratitude as relational process	Emerges through reciprocity, social bonds, and interpersonal attunement	Gratitude letters or interpersonal acknowledgment exercises	Social reinforcement and prosocial reciprocity	Relational gratitude is often operationalized through solitary practices
Gratitude as moral virtue	Ethical orientation involving humility, recognition, and reciprocity	Rarely intervention-based; mostly conceptual discussion	Moral cultivation and ethical reflection	Limited translation into empirical or experimental paradigms
Gratitude as existential orientation	Appreciation grounded in existence, interconnectedness, or being alive	Occasionally contemplative or mindfulness-based practices	Receptive awareness and existential attunement	Largely absent from mainstream gratitude interventions and measurement tools
Gratitude as spiritual/contemplative practice	Non-contingent mode of receptivity, presence, and transcendence	Meditation, contemplative awareness, spiritual reflection	Expanded awareness and transpersonal experience	Poorly integrated into dominant psychological and neuroscientific research paradigms

## 6 Gratitude in neuroscience

### 6.1 Current neuroscience paradigms

The neuroscientific studies reviewed operationalize gratitude through several distinct approaches. Experimental fMRI studies commonly use imagined or interactive scenarios in which participants receive intentional help, protection, or pain reduction (Fox et al., 2015b; Yu et al., 2017, 2018). These paradigms associate gratitude-related appraisals with neural systems involved in valuation, reward, mentalizing, and social cognition, including the medial prefrontal cortex, anterior cingulate cortex, temporoparietal junction, and ventral striatum.

Longitudinal studies examine whether gratitude practices influence subsequent neural processing. Gratitude writing has been associated with changes in medial prefrontal activity (Kini et al., 2016). After three weeks of gratitude journaling, Karns et al. (2017) observed increased neural responses to gains benefiting charity relative to oneself. These findings suggest that gratitude practice may alter the valuation of prosocial outcomes, although they do not directly demonstrate changes in the neural experience of gratitude.

Psychophysiological research provides preliminary evidence of coordination between gratitude-related states and autonomic activity. McCraty et al. (1995) found that self-induced appreciation produced cardiac patterns distinct from those observed during anger, although the study examined appreciation rather than clearly differentiated gratitude and requires independent replication. Kyeong et al. (2017) found that a brief gratitude meditation was associated with lower heart rate than a resentment condition, differences in brain–heart coupling, and changes in functional connectivity within emotion- and motivation-related networks.

Structural imaging offers a complementary dispositional perspective. Zahn et al. (2014) and Liu et al. (2018) identified associations between gratitude proneness or trait gratitude and regional grey-matter volume. Because these studies were cross-sectional, they neither establish that gratitude causes structural change nor identify a distinctive neuroanatomical signature of gratitude.

These paradigms provide evidence about the neural correlates of interpersonal benefit appraisal, prosocial valuation, gratitude-related practice, and dispositional tendencies. Their findings cannot automatically be generalized to existential, ecological, or contemplative gratitude, and processes shared with appreciation, positive affect, social cognition, emotion regulation, and prosociality remain important alternative explanations. The central consequence for gratitude neuroscience is therefore one of interpretive scope: conclusions about its neural mechanisms depend on the particular form of gratitude elicited or measured.

### 6.2 Toward embodied and ecologically valid gratitude paradigms

Expanding gratitude neuroscience requires paradigms that elicit and measure a broader range of grateful experiences while remaining compatible with scanner constraints. Validated audiovisual stimuli could evoke gratitude arising through nature, beauty, interconnectedness, or meaningful human experience. Autobiographical scripts could be developed from participants’ own gratitude narratives and presented alongside matched neutral or interpersonal-benefit conditions. Prerecorded relational interactions could similarly examine gratitude emerging through expressions of care, shared vulnerability, or interpersonal presence.

Ecological momentary assessment could provide individualized cues derived from gratitude experienced in everyday life. Participants could record the context, object, intensity, bodily qualities, and meaning of each experience; selected episodes could then be reconstructed for presentation during fMRI. This approach would preserve greater personal and contextual relevance while allowing standardized comparison conditions and repeated measurement.

Embodied dimensions of gratitude could be examined by combining neuroimaging with cardiac, respiratory, and electrodermal recording. Trial-by-trial measures should distinguish gratitude from related states such as appreciation, awe, positive affect, indebtedness, connectedness, and meaning. Together, these methods would enable researchers to compare the neural and autonomic processes associated with interpersonal, contemplative, existential, and ecological forms of gratitude without assuming that they constitute a single homogeneous state.

## 7 Discussion and future directions

This review contributes by specifying the limits of treating gratitude as a unitary construct. Benefit-focused paradigms have generated valuable evidence concerning interpersonal appraisal, prosociality, and well-being, but gratitude may also arise through awe, contemplation, adversity, nature, and broader forms of interconnectedness. The proposed reframing of gratitude as relational participation offers a way to examine these experiences without assuming that they all depend on receiving an intentional benefit from an identifiable benefactor.

A first priority is to determine whether interpersonal, existential, contemplative, and ecological gratitude share common processes or constitute partly distinct experiential forms. Comparative and factorial studies could vary benefactor presence, benefit intentionality, recipient need, adversity, and relational or environmental context. Such designs could establish which features are common across grateful experiences and which depend on the circumstances through which gratitude arises.

A second priority is to identify mechanisms, boundary conditions, and outcomes. Research should examine how benefit appraisal, meaning-making, awe, connectedness, bodily regulation, and self-transcendence contribute to different forms of gratitude. It should also test whether their effects vary according to individual differences, cultural background, relational context, or exposure to adversity. Rather than asking only whether gratitude is beneficial, studies could investigate which forms promote well-being, resilience, cooperation, meaning, or environmental responsibility, for whom, and under what conditions.

This programme requires state-sensitive measurement extending beyond gratitude intensity. Measures could assess benefactor and benefit perception, bodily sensations, connectedness, meaning, awe, indebtedness, emotional complexity, and action tendencies. Qualitative and micro-phenomenological approaches may identify experiential features not captured by existing scales, while ecological momentary assessment can examine how gratitude unfolds across everyday interpersonal, contemplative, and environmental contexts. Intervention research should likewise distinguish the forms of gratitude being cultivated and include comparison conditions capable of isolating their proposed mechanisms.

The implications extend across several levels. At the neural and individual levels, differentiating forms of gratitude could clarify their respective relationships with valuation, social cognition, self-referential processing, bodily regulation, well-being, and resilience. Culturally, it may reveal experiences that are obscured when gratitude is understood primarily through interpersonal benefit exchange. In organizations, distinguishing gratitude for discrete benefits from appreciation, relational presence, and collective belonging could support more context-sensitive practices without reducing gratitude to obligatory positivity or reciprocity. At the societal level, ecological and collective forms of gratitude may inform research on solidarity, care for shared institutions, and responsibility toward the natural environment. The broader aim is therefore not merely to expand the category of gratitude, but to develop more precise explanations of its forms, mechanisms, boundaries, and consequences.

## 8 Conclusion

This review identifies a conceptual–operational mismatch within the gratitude literature examined: gratitude is conceptualized in interpersonal, existential, contemplative, transpersonal, and ecological terms, yet influential empirical paradigms often focus on reflecting upon benefits, benefactors, and positive experiences. Recognizing the specific form of gratitude elicited or measured is essential for interpreting findings and defining their scope. By reframing gratitude as a possible mode of relational participation, this review offers a foundation for research that distinguishes its forms, mechanisms, contexts, and consequences.

## References

[ref1] Algoe S. B. (2012). Find, remind, and bind: the functions of gratitude in everyday relationships. Soc. Personal. Psychol. Compass 6, 455–469. doi: 10.1111/j.1751-9004.2012.00439.x

[ref2] Algoe S. B. Haidt J. Gable S. L. (2008). Beyond reciprocity: gratitude and relationships in everyday life. Emotion 8, 425–429. doi: 10.1037/1528-3542.8.3.425, 18540759 PMC2692821

[ref3] Bartlett M. Y. DeSteno D. (2006). Gratitude and prosocial behavior: helping when it costs you. Psychol. Sci. 17, 319–325. doi: 10.1111/j.1467-9280.2006.01705.x, 16623689

[ref4] Büssing A. Recchia D. R. Baumann K. (2018). Validation of the gratitude/awe questionnaire and its association with disposition of gratefulness. Religion 9:117. doi: 10.3390/rel9040117

[ref7] Emmons R. A. Kneezel T. T. (2005). Giving thanks: spiritual and religious correlates of gratitude. J. Psychol. Christ. 24, 140–148.

[ref8] Emmons R. A. McCullough M. E. (2003). Counting blessings versus burdens: an experimental investigation of gratitude and subjective well-being in daily life. J. Pers. Soc. Psychol. 84, 377–389. doi: 10.1037/0022-3514.84.2.377, 12585811

[ref9] Emmons R. A. Shelton C. M. (2002). “Gratitude and the science of positive psychology,” in Handbook of Positive Psychology, eds. Snyder C. R. Lopez S. J. (New York: Oxford University Press), 459–471.

[ref10] Fox G. R. Araujo H. F. Metke M. J. Shafer C. Damasio A. (2015a). How does the effort spent to hold a door affect verbal thanks and reciprocal help? Front. Psychol. 6:article 1737. doi: 10.3389/fpsyg.2015.01737, 26617559 PMC4641909

[ref11] Fox G. R. Kaplan J. Damasio H. Damasio A. (2015b). Neural correlates of gratitude. Front. Psychol. 6:article 1491. doi: 10.3389/fpsyg.2015.01491, 26483740 PMC4588123

[ref12] Fox G. R. Shah K. Brownell K. M. (2026). A grateful space: understanding a range of gratitude experiences. J. Posit. Psychol. 21, 89–101. doi: 10.1080/17439760.2024.2427595

[ref13] Gouldner A. W. (1960). The norm of reciprocity: a preliminary statement. Am. Sociol. Rev. 25, 161–178. doi: 10.2307/2092623

[ref14] Hirshberg M. J. Goldberg S. B. Schaefer S. M. Flook L. Findley D. Davidson R. J. (2018). Divergent effects of brief contemplative practices in response to an acute stressor: a randomized controlled trial of brief breath awareness, loving-kindness, gratitude or an attention control practice. PLoS One 13:e0207765. doi: 10.1371/journal.pone.0207765, 30540772 PMC6291192

[ref15] Hlava P. Elfers J. Offringa R. (2014). A transcendent view of gratitude: the transpersonal gratitude scale. Int. J. Transpers. Stud. 33, 1–14.

[ref16] Jans-Beken L. Wong P. T. P. (2021). Development and preliminary validation of the existential gratitude scale (EGS). Couns. Psychol. Q. 34, 72–86. doi: 10.1080/09515070.2019.1656054

[ref17] Karns C. M. Moore W. E. Mayr U. (2017). The cultivation of pure altruism via gratitude: a functional MRI study of change with gratitude practice. Front. Hum. Neurosci. 11:599. doi: 10.3389/fnhum.2017.00599, 29375336 PMC5770643

[ref18] Kini P. Wong J. McInnis S. Gabana N. Brown J. W. (2016). The effects of gratitude expression on neural activity. NeuroImage 128, 1–10. doi: 10.1016/j.neuroimage.2015.12.040, 26746580

[ref19] Kyeong S. Kim J. Kim D. J. Kim H. E. Kim J.-J. (2017). Effects of gratitude meditation on neural network functional connectivity and brain–heart coupling. Sci. Rep. 7:5058. doi: 10.1038/s41598-017-05520-9, 28698643 PMC5506019

[ref20] Lane J. Anderson N. H. (1976). Integration of intention and outcome in moral judgment. Mem. Cogn. 4, 1–5. doi: 10.3758/BF03213247, 21286951

[ref21] Liu G. Zeng G. Wang F. Rotsthein P. Peng K. Sui J. (2018). Praising others differently: neuroanatomical correlates to individual differences in trait gratitude and elevation. Soc. Cogn. Affect. Neurosci. 13, 1225–1234. doi: 10.1093/scan/nsy093, 30351412 PMC6277740

[ref22] McCraty R. Atkinson M. Tiller W. A. Rein G. Watkins A. D. (1995). The effects of emotions on short-term power spectrum analysis of heart rate variability. Am. J. Cardiol. 76, 1089–1093. doi: 10.1016/S0002-9149(99)80309-9, 7484873

[ref23] McCullough M. E. Emmons R. A. Tsang J. A. (2002). The grateful disposition: a conceptual and empirical topography. J. Pers. Soc. Psychol. 82, 112–127. doi: 10.1037/0022-3514.82.1.112, 11811629

[ref24] McCullough M. E. Kilpatrick S. D. Emmons R. A. Larson D. B. (2001). Is gratitude a moral affect? Psychol. Bull. 127, 249–266. doi: 10.1037/0033-2909.127.2.249, 11316013

[ref25] Nowak M. A. Roch S. (2007). Upstream reciprocity and the evolution of gratitude. Proc. R. Soc. B Biol. Sci. 274, 605–610. doi: 10.1098/rspb.2006.0125, 17254983 PMC2197219

[ref27] Seligman M. E. P. Steen T. A. Park N. Peterson C. (2005). Positive psychology progress: empirical validation of interventions. Am. Psychol. 60, 410–421. doi: 10.1037/0003-066X.60.5.410, 16045394

[ref28] Tesser A. Gatewood R. Driver M. (1968). Some determinants of gratitude. J. Pers. Soc. Psychol. 9, 233–236. doi: 10.1037/h0025905, 5666969

[ref30] Watkins P. C. (2013). Gratitude and the Good Life: Toward a Psychology of Appreciation. Dordrecht: Springer.

[ref31] Watkins P. C. Woodward K. Stone T. Kolts R. L. (2003). Gratitude and happiness: development of a measure of gratitude, and relationships with subjective well-being. Soc. Behav. Pers. 31, 431–452. doi: 10.2224/sbp.2003.31.5.431

[ref32] Wong P. T. P. (2011). Positive psychology 2.0: towards a balanced interactive model of the good life. Can. Psychol. 52, 69–81. doi: 10.1037/a0022511

[ref34] Wood A. M. Froh J. J. Geraghty A. W. A. (2010). Gratitude and well-being: a review and theoretical integration. Clin. Psychol. Rev. 30, 890–905. doi: 10.1016/j.cpr.2010.03.005, 20451313

[ref35] Yu H. Cai Q. Shen B. Gao X. Zhou X. (2017). Neural substrates and social consequences of interpersonal gratitude: intention matters. Emotion 17, 589–601. doi: 10.1037/emo0000258, 27936814

[ref36] Yu H. Gao X. Zhou Y. Zhou X. (2018). Decomposing gratitude: representation and integration of cognitive antecedents of gratitude in the brain. J. Neurosci. 38, 4886–4898. doi: 10.1523/JNEUROSCI.2944-17.2018, 29735557 PMC6596125

[ref37] Zahn R. Garrido G. Moll J. Grafman J. (2014). Individual differences in posterior cortical volume correlate with proneness to pride and gratitude. Soc. Cogn. Affect. Neurosci. 9, 1676–1683. doi: 10.1093/scan/nst158, 24106333 PMC4221203

