# Supplementary material for: What is gratitude research missing? Bridging the gap between conceptualization, measurement, and intervention
Source: Front Psychol. 2026 Oct 15;17:1910738. doi: 10.3389/fpsyg.2026.1910738 (PMC13717311; doi:10.3389/fpsyg.2026.1910738)
Supplement: Supplementary file 1 [file Table_1.DOCX]

SUPPLEMENTARY TABLE S1 Comparative Overview of Selected Gratitude Intervention Paradigms.

**Note.** Typical implementations and reported effects refer to the cited studies and should not be interpreted as universal characteristics of each intervention category. Proposed mechanisms summarize intervention rationales or theoretically informed interpretations and have not necessarily been tested as mediators. Likely implementation demands represent the authors’ comparative assessment. Where adherence or persistence was not directly assessed, this is indicated rather than inferred.
